# Serotonin (5-HT) Affects Expression of Liver Metabolic Enzymes and Mammary Gland Glucose Transporters during the Transition from Pregnancy to Lactation

**DOI:** 10.1371/journal.pone.0057847

**Published:** 2013-02-28

**Authors:** Jimena Laporta, Tonia L. Peters, Kathryn E. Merriman, Chad M. Vezina, Laura L. Hernandez

**Affiliations:** 1 Department of Dairy Science, University of Wisconsin-Madison, Madison, Wisconsin, United States of America; 2 Department of Comparative Biosciences, School of Veterinary Medicine, University of Wisconsin-Madison, Madison, Wisconsin, United States of America; Hosptial Infantil Universitario Niño Jesús, CIBEROBN, Spain

## Abstract

The aim of this experiment was to demonstrate the ability of feeding serotonin (5-HT; 5-hydroxytryptamine) precursors to increase 5-HT production during the transition from pregnancy to lactation and the effects this has on maternal energy metabolism in the liver and mammary gland. Pregnant rats (n = 45) were fed one of three diets: I) control (CON), II) CON supplemented with 0.2% 5-hydroxytryptophan (5-HTP) or III) CON supplemented with 1.35% L-tryptophan (L-TRP), beginning on d13 of pregnancy through d9 of lactation (d9). Serum (pre and post-partum), milk (daily), liver and mammary gland tissue (d9) were collected. Serum 5-HT was increased in the 5-HTP fed dams beginning on d20 of gestation and remained elevated through d9, while it was only increased on d9 in the L-TRP fed dams. 5-HT levels were increased in mammary gland and liver of both groups. Additionally, 5-HTP fed dams had serum and milk glucose levels similar to the CON, while L-TRP had decreased serum (d9) and milk glucose (all dates evaluated). Feeding 5-HTP resulted in increased mRNA expression of key gluconeogenic and glycolytic enzymes in liver and glucose transporters 1 and 8 (GLUT-1, -8) in the mammary gland. We demonstrated the location of GLUT-8 in the mammary gland both in the epithelial and vascular endothelial cells. Finally, phosphorylated 5′ AMP-activated protein kinase (pAMPK), a known regulator of intracellular energy status, was elevated in mammary glands of 5-HTP fed dams. Our results suggest that increasing 5-HT production during the transition from pregnancy to lactation increases mRNA expression of enzymes involved in energy metabolism in the liver, and mRNA abundance and distribution of glucose transporters within the mammary gland. This suggests the possibility that 5-HT may be involved in regulating energy metabolism during the transition from pregnancy to lactation.

## Introduction

The transition from pregnancy to lactation is a critical period for most mammalian species. Glucose is used as a fuel for the animal and fetus and also as a precursor for lactose formation in the mammary gland [1]. At the onset of lactation, maternal tissues, particularly the liver and the mammary glands, undergo numerous adaptations to support milk synthesis. It is during the transition period, particularly in dairy cattle, as well as other mammalian species, that there is a decrease in feed intake leading to a severe negative energy balance (NEB) during the early lactation period [2]. The ability of the mother to overcome NEB during this period is critical to the ability of the lactation to proceed successfully. These adaptations are mediated by changes in hormones and metabolites, increased hepatic gluconeogenesis, and decreased peripheral tissue glucose utilization to supply glucose for the mammary gland [1]. Enhancement of pathways related to glucose production during the transition from pregnancy to lactation, are critical for minimizing glycogen depletion in the liver, which circumvents metabolic disorders such as ketosis [2].

Recent studies suggest a role for the monoamine serotonin (5-hydroxytryptamine, 5-HT) in hepatic glucose metabolism [3]. 5-HT is synthesized in a variety of peripheral tissues, including the gut, bone and mammary gland [4]–[6]. It is derived from the amino acid L-tryptophan (L-TRP), which is hydroxylated to 5-hydroxy-L-tryptophan (5-HTP) by tryptophan hydroxylase-1 (TPH1), the rate-limiting step in 5-HT biosynthesis [7]. 5-HT acts through more than 15 known receptor subtypes (5-HTR) and its synthesis and subsequent degradation is controlled by a 5-HT reuptake transporter (SERT), making the 5-HT system very complex [8]. Numerous studies have demonstrated the role of mammary synthesized 5-HT on various aspects of mammary gland development and lactation [4], [9]–[11]. Furthermore, various 5-HTR subtypes were identified within the mammary gland and appear to regulate different aspects of mammary gland homeostasis [11]–[13]. Additionally, TPH1, the rate-limiting enzyme in 5-HT synthesis, is expressed in the liver and various 5-HTR subtypes have been identified [14]–[15]. 5-HT is thought to mediate effects such as hepatic regeneration [16] and glucose and insulin homeostasis [3], [17]. Studies have shown that 5-HT is involved in liver glucose uptake mechanisms [18], [19], [20], and glycogen metabolism [14]. Coelho et al. [21] demonstrated the ability of 5-HT to control hepatic glycolysis through regulation of hepatic phosphofructokinase (PFK) activity. To date little is known about the involvement of 5-HT in glucose homeostasis during the transition from pregnancy to lactation.

Given the role of 5-HT in mammary gland homeostasis, and its involvement in glucose metabolism, we set out to determine the role of 5-HT in glucose metabolism during the transition from pregnancy to lactation. Our objective was to increase endogenous peripheral (non-neuronal) 5-HT levels, via feeding supplemental L-TRP or 5-HTP, two known precursors for 5-HT synthesis, and to determine the contribution of increased 5-HT in the regulation of known enzymes and transporters involved in energy metabolism in the mammary gland and liver during the transition from pregnancy to lactation.

## Materials and Methods

### Ethics Statement

The animal experiments were approved by the College of Agriculture and Life Sciences Animal Care and Use Committee at the University of Wisconsin-Madison. Their guidelines for the care and use of the animals were strictly followed for all experiments. The protocol number assigned for these experiments was A01473 to Dr. Laura L. Hernandez and was entitled Hormone and Genetic Studies in Rodents.

### Animal Care and Experiment Design

Forty-five timed pregnant Sprague-Dawley rats (Harlan Laboratory) approximately 5 months of age were utilized and maintained throughout the experiment in the Biotron, a controlled environmental facility for biological research at the University of Wisconsin-Madison. Rats were maintained at 25°C, constant humidity (50–60%), and a 12 h light/dark cycle with free access to food and water. Animals were randomly assigned to individual cages and fed one of three diets beginning on d13 of pregnancy and ending on d9 of lactation: control (CON, TestDiet® AIN-93G Growth Purified Diet, n = 15), 5-HTP (0.2% of total CON diet, n = 15) and L-TRP (1.35% of total CON diet, n = 15) with the goal of increasing endogenous 5-HT production during the transition period (d13 of pregnancy – d9 of lactation). Litter size was standardized to 10 pups per dam on the day of parturition.

### Data and Sample Collection

Serum samples were harvested from blood collected from the pedal vein of all animals on d20 of gestation, and d1 and d9 of lactation. For milking, animals were injected with 0.6 units of oxytocin (i.m.) to stimulate milk let down. Milk samples were collected on d1, 5 and 9 of lactation by vacuum pump [22]. Litter weights and milk yield were recorded daily, with milk yield being estimated using the weigh-suckle-weigh method once daily [22]. On d9 of lactation all dams were euthanized and liver and mammary gland (right #4 gland) tissue were obtained for RNA and total protein isolation. Tissue was stored at −80°C until used. The left #4 mammary gland was fixed in 4% paraformaldehyde overnight at 4°C and then transferred to 70% ethanol. Tissue was subsequently embedded in paraffin and sectioned (5 *µ*m) for histological evaluation.

### Protein Isolation

Protein was isolated from mammary gland and liver tissues using radioimmunoprecipitation assay (RIPA) buffer with protease inhibitors (0.58 mM PMSF, 1 mM sodium orthovandate and 2 *µ*g/ml aprotinin). Total protein concentrations were determined using the bicinchoninic acid (BCA) protein assay (Pierce Chemicals). Liver and mammary gland 5-HT levels were measured by ELISA (enzyme immunoassay (EIA) kit Enzo life Sciences®, #ADI-900-175). The detection limit of the assay was 0.49 ng/ml and the intra-assay coefficient of variation (CV) was less than 1.7% for both tissues.

### Western Blotting

Mammary gland protein extracts (30 *µ*g) were separated by electrophoresis on a 7.5% SDS-polyacrylamide gel and transferred for 1 h at 4°C onto polyvinylidene difluoride membrane (Millipore #IPVH00010). Membranes were blocked with 5% BSA for 1 h and probed overnight at 4°C with 1∶1,000 phosphorylated AMP-activated protein kinase (p-AMPK-Thr172; Cell Signaling Technology #2535). Membranes were then incubated for 1 h at room temperature with horseradish peroxidase-conjugated secondary antibody (1∶10,000 donkey anti-rabbit IgG Santa Cruz, Biotech #sc-2305) and 1∶10,000 StrepTactin-HRP conjugate (Bio-Rad #161-0381) for chemiluminescent detection. Protein bands were detected using Immun-Star™ WesternC™ Kit (BioRad #170-5070) and visualized using the Chemidoc XRS system (BioRad #1708070). The same membrane was then stripped twice and re-probed with 1∶1,000 total-AMPK (Cell Signaling Technology #2532) and then 1∶5,000 β-actin (Sigma #A5441) and horseradish peroxidase-conjugated donkey anti-rabbit IgG (1∶10,000, Santa Cruz #sc-2305) or donkey anti-mouse IgG (1∶10,000, Santa Cruz #sc-2314) were used as secondary antibodies as appropriate. Image processing and protein band quantification was done using QuantityOne (v.4.6.9) Analysis Software (BioRad).

### Serum and Milk Assays

Serum 5-HT levels were measured by ELISA using a 5-HT EIA kit (Enzo life Sciences® #ADI-900-175) according to the manufactureŕs instructions. The detection limit of the assay was 0.43 ng/ml and the intra-assay CV was 5.2%. Serum insulin concentrations were measured with the ultra-sensitive Rat ELISA Insulin kit (Crystal Chem, Inc. #90060) according to the manufactureŕs instructions. The detection limit was 0.047 ng/ml and the inter- and intra-assay CV’s were less than 6.5% and 5.7%, respectively. Glucose concentrations were measured in serum and milk samples by the glucose oxidase-peroxidase assay that is specific for glucose [23]. Serum was diluted 1∶20 and milk 1∶100 in order to detect glucose concentrations within the constraints of the assay. The detection limits for the glucose assay were 0.6 mg/dl and the inter- and intra-assay CV’s were 2% for serum, and 4.5% for milk glucose assays, respectively. Milk lactose was measured using a lactose assay kit (BioVision, #K624-100) according to the manufactureŕs instructions. Milk was diluted 1∶100 in order to detect lactose concentrations within the constraints of the assay. The detection limit of the lactose assay was 27 mmol, and inter and intra-assay CV’s were 5.6%, and 7.5%, respectively.

### Quantitative Real-time RT-PCR (qPCR)

Total RNA was isolated from liver and mammary tissue using TRI-Reagent® (Molecular Research). mRNA concentration and absorbance ratios were determined using a Nanodrop spectrophotometer (ND-1000; Nanodrop Technologies). One *µ*g of total RNA was reversed transcribed using iScript™ reverse transcription supermix for RT-qPCR Kit (BioRad #1708841) and diluted (1∶5) in deionized water. qPCR was conducted using the CFX96 Touch Real-Time PCR Detection System (Bio-Rad) using SSoFast™ EvaGreen® Supermix (BioRad # 1725203) per manufacturer’s instructions. Amplification efficiencies of primers were evaluated and were within a range of 95 and 105% efficiency, and primer specificity was assessed by the presence of a single temperature dissociation peak. A negative RT sample and a water control were run on all plates. Primer sequences are listed in Table 1. Ribosomal S15 was used as the housekeeping gene, as it illustrated consistent expression in all treatment groups, and analysis was conducted using the 2^−ΔΔCt^ method with the control fed animals serving as the basis of comparison [24].

**Table 1:** Primers utilized for qPCR analysis.

Primer	Forward (5′→3′)	Reverse (5′→3′)
***Glucose transporters***		
GLUT-1	TGCAGTTCGGCTATAACACC	ACACCTCCCCCACATACATG
GLUT-8	GCTTGGTCCTAAGCAACTGG	CTTCCTGGACTCACCTGACC
***Gluconeogenic enzymes***		
PC	CCCAAGCCTCTCAACAGAAG	GCCATTGCAGGTAGTGTGTG
PDK4	CGTCGTCTTGGGAAAAGAAG	CGTGAATTGTCCATCACAGG
PCK1	GTGTCCCCCTTGTCTACGAA	GACCTTGCCCTTATGCTCTG
FPK1	GCATCACCAACCTGTGTGTC	TCTTGCCTTCCTTCACCAGT
***5-HT synthesis***		
TPH1	CAAGGAGAACAAAGACCATTC	CGCAGTCCACAAAAATCT
***Housekeeping***		
S15	CTTCCGCAAGTTCACCTACC	GTTGTACACACCCACCAT

* GLUT-1 = glucose transporter-1, GLUT-8 = glucose transporter-8, PC = pyruvate carboxylase, PDK4 = pyruvate dehydrogenase kinase, isozyme 4, PCK1 = phosphoenolpyruvate carboxykinase-1 PFK1 = phosphofructokinase-1, TPH1 = tryptophan hydroxylase-1, S15 = ribosomal protein S15. 60°C was used as an annealing temperature for all primers.

### Histological Examination of Mammary Tissue

Serial sectioned mammary glands (5 µM) were used for histological examination of mammary tissue. Sections were stained with hematoxylin and eosin (H&E). Alveolar lumen diameter was calculated using ImageJ 1.46r Software (National Institutes of Health). Five alveoli were measured per animal (5 per treatment group) under 20× objective lens magnification. Immunostaining for glucose transporter- 1 (GLUT-1) and glucose transporter- 8 (GLUT-8) were performed using rabbit polyclonal anti-GLUT-1 and anti-GLUT-8 (Millipore #071401 and 071407, respectively) at a 1∶100 dilution overnight at 4°C followed by 1∶500 goat anti-rabbit secondary antibody (Vectastain) for 1 h at room temperature. Sections with no primary antibody were included for both antibodies as negative controls. Vectastain ABC *Elite* kit (Vector Laboratories #PK-6101) was used for immunostaining per manufacturer’s instructions and the DAB Kit (Vector Laboratories #SK-4100) was used for visualization according to manufacturer’s instructions. Images were captured (20X magnification) with a Nikon 80i microscope fitted with a Nikon DS-Fi1 digital RGB camera using Nikon NIS Elements software.

### Immunofluorescence

Mammary sections were incubated with rabbit polyclonal anti-GLUT-8, (1∶100 Millipore #071407) in combination with either rat anti-mouse, cytokeratin 8 (K8) which labels luminal epithelial cells (Troma 1, 1∶100 Developmental Studies Hybridoma Bank, University of Iowa), smooth muscle actin NCL-SMA that labels myoepithelium (SMA, 1∶250 Novocastra, Leica Biosystems #6000040) primary antibodies or fluorescein-labeled griffonia (bandeiraea) simplicifolia lectin I, isolectin B4 that labels vascular endothelium (GS1, 1∶50 Vector Laboratories #FL-1201) overnight at 4^o^ C. Secondary antibodies were diluted as follows: 1∶250 DyLight^TM^549-conjugated anti-rabbit IgG (Jackson Immuno Research #111-507-003), 1∶250 DyLight^TM^488-conjugated anti-rat IgG (Jackson Immuno Research #112-496-003), and 1∶250 DyLight™ 488-conjugated anti-mouse IgG (Jackson Immuno Research #115-487-003) for GLUT-8, GS1 and SMA, respectively, and incubated for 1 h at room temperature. Additionally, mammary gland sections were incubated with rabbit polyclonal anti-GLUT-1, (1∶100, Millipore #071401) primary antibody and tested for co-localization with GS1 and K8, as described above. Nuclei were visualized with 4′,6-diamidino-2-phenylindole (DAPI, 1∶1,000). Images were captured (20X magnification) with a Nikon E600 Eclipse fluorescence microscope fitted with a Nikon DS-Fi1 RGB camera and processed and merged to detect co-localization patterns using Nikon NIS Elements software.

### Statistical Analyses

Data were analyzed with Prism for Macintosh, version 5.0b (GraphPad Software, San Diego, CA) for all experiments. Gene expression, tissue protein, and maternal body weights were analyzed by a one-way ANOVA, with a non-parametric Kruskal-Wallis Test and Dunn’s *post-hoc* test due to the determination of samples having unequal variances by Bartlett’s and Brown-Forsythe tests. Alveolar diameter was analyzed by a one-way ANOVA with a Tukey’s *post-hoc* test to make comparisons between treatments. Serum 5-HT, insulin and glucose, milk lactose and glucose, and milk yield data were analyzed by 2-way ANOVA with time and treatment included in the model and Bonferroni’s *post-hoc* test for multiple comparisons between groups. For all analyses, differences between means were considered significant at *P*≤0.05. All values are reported as means ± SEM.

## Results

### Maternal Milk Yield and Offspring Growth

**Figure 1:**
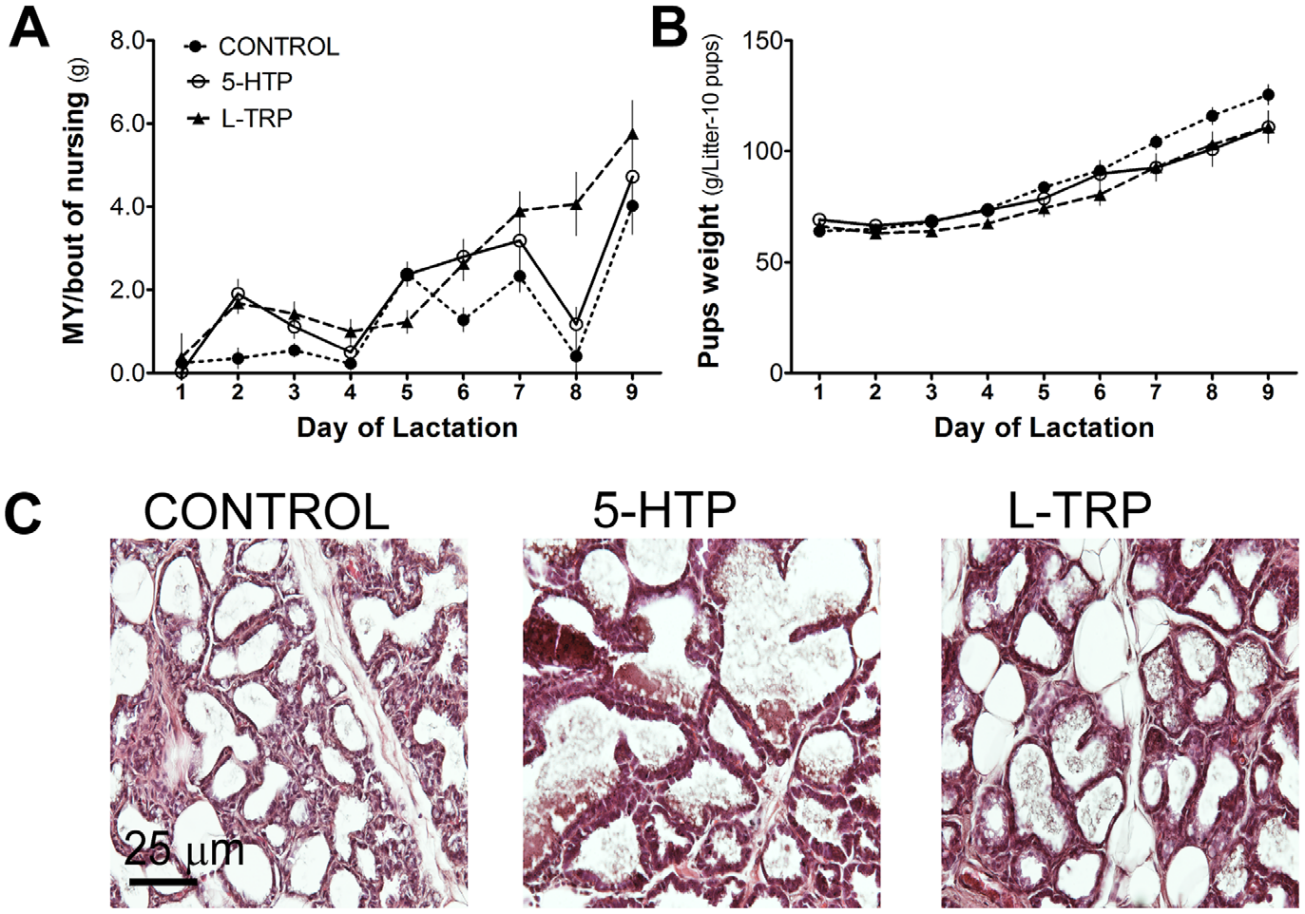
Feeding diets enriched in 5-HTP and L-TRP does not affect milk yield, pup growth or mammary gland structure. (A) Milk yield (MY) estimated by weigh-suckle-weigh method. (B) Daily litter weights (g, d1 to d9 of lactation). (C) Hematoxylin and eosin stained sections of mammary glands collected on d9 of lactation (20X magnification), in Sprague-Dawley rats fed CON, 5-HTP, and L-TRP.

In high concentrations, 5-HT is known to induce mammary gland involution, therefore it was pertinent to determine the effects of increasing endogenous 5-HT production on milk synthesis as well as mammary gland structure [4], [25]. Milk yield was equal among all groups, until d8 and d9 of lactation, in which it increased in L-TRP animals (*P*<0.05, Figure 1a). Pup litter weights were not different between treatments throughout the experiment (Figure 1b). Additionally, maternal body weights were not different between cohorts (297.5±1.97, 278.8±3.44, and 276.3±4.16 means ± SEM, for CON, 5-HTP and L-TRP, respectively). Hematoxylin and eosin staining of mammary sections were used to confirm the integrity and structure of the mammary tissues (i.e., absence of vacuoles, decreased luminal space), which was similar among treatments at d9 of lactation per examination by a pathologist (Figure 1c). Alveolar lumen diameter was not significantly different between treatments (328.5±15.44, 367.5±27.13, and 329.1±17.45 means ± SEM, for CON, 5-HTP, and L-TRP, respectively).

### Evaluation of 5-HT in Response to Feeding Supplemental 5-HTP and L-TRP

**Figure 2:**
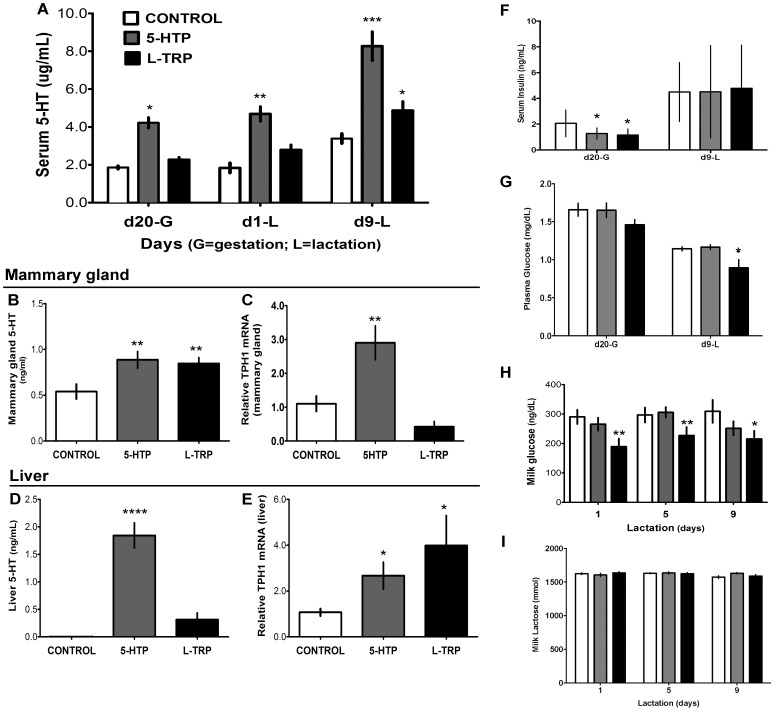
Effects of feeding diets enriched in 5-HTP and L-TRP on serum 5-HT, insulin and glucose in rats. Serum 5-HT concentrations on d20 of gestation (d20-G), d1 and d9 of lactation (d1-L, d9-L) (A). 5-HT protein concentration and mRNA expression of TPH1 in the mammary gland (B-D) and liver (E-G) on d9-L. Serum insulin (H) and plasma glucose (I) concentrations on d20-G and d9-L, milk glucose concentrations at d1, d5 and d9 of lactation (J), in dams fed CON, 5-HTP, and L-TRP diets, and milk lactose concentrations at d1, d5, and d9 of lactation (I), in dams fed CON, 5-HTP, and L-TRP diets. Data are represented as mean ± SEM. Single asterisk (*) indicate statistical significance at *P*<0.05, two asterisks (**) at *P*<0.01, three (***) at *P*<0.001 and four (****) at *P*<0.0001.

We set out to determine if feeding precursors for 5-HT would not only increase circulating concentrations of 5-HT, but also whether the synthesis of 5-HT in the mammary gland and liver were also increased. It is a well-known fact that 5-HT is synthesized and secreted by various tissues throughout the body in addition to the circulating platelets, with the gut synthesizing approximately 95% of circulating 5-HT [26]. Serum 5-HT levels were greater in rats fed 5-HTP than CON for all times evaluated, and were only elevated in the L-TRP diet on d9 of lactation (*P*<0.01, Figure 2a). 5-HT concentrations were increased in mammary gland tissue of both 5-HTP and L-TRP dams compared to the CON (*P*<0.009, Figure 2b). In the liver, 5-HT was undetectable in the CON, but was increased in both the 5-HTP and L-TRP fed dams, with the 5-HTP fed dams exhibiting the highest levels of 5-HTP (*P*<0.04, Figure 2d). mRNA expression of TPH1 was increased in the 5-HTP group in both mammary gland and liver, and only in the liver of the L-TRP group (*P*<0.042, Figures 2c, e).

### Maternal Glucose and Insulin Concentrations

In attempt to determine the effects of increased 5-HT levels in the circulation on glucose metabolism, we measured circulating insulin and glucose concentrations and milk lactose and glucose concentrations. Maternal serum insulin was decreased in both treatment groups compared to the CON on d20 of gestation (*P*<0.03) but it was similar to CON animals on d9 of lactation (*P*<0.001, Figure 2f). Maternal serum glucose concentrations decreased from d20 of gestation to d9 of lactation in all groups, with the L-TRP fed dams exhibiting significantly lower glucose concentrations than 5-HTP and CON animals (*P*<0.001, Figure 2g). Maternal milk glucose concentrations were decreased in the L-TRP group compared to the CON on all dates evaluated, but no differences were detected between the CON and the 5-HTP fed dams (*P*<0.001, Figure 2h). Milk lactose was equal among all groups (*P*>0.05, Figure 2i).

### mRNA Expression of Hepatic Markers of Energy Metabolism

**Figure 3:**
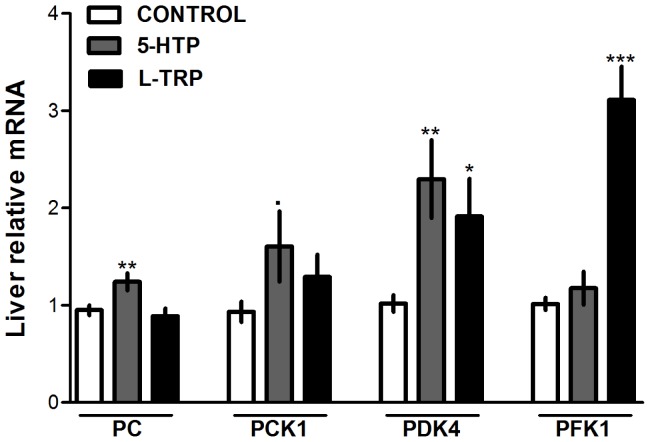
Liver mRNA expression of gluconeogenic and glycolytic enzymes. Liver mRNA expression of dams on d9 of lactation of pyruvate carboxylase (PC), phosphoenolpyruvate carboxykinase-1 (PCK1), pyruvate dehydrogenase kinase, isozyme 4 (PDK4), and phosphofructokinase-1 (PFK1) in dams fed CON, 5-HTP, and L-TRP diets. Data are represented as mean ± SEM. Single asterisk (*) indicate statistical significance at *P*<0.05, two asterisks (**) at *P*<0.01, three (***) at *P*<0.001.

Hepatic regulation of energy metabolism is critical for the support of glucose homeostasis during times of low energy supply, as seen during the transition from pregnancy to lactation [27]. Liver mRNA expression of key enzymes involved in energy metabolism was evaluated in this study. Maternal liver mRNA expression of pyruvate carboxylase (PC) was increased only in the dams consuming the 5-HTP diet (*P* = 0.0087, Figure 3), and phosphoenolpyruvate carboxykinase 1 (PCK1) tended to increase in those animals as well (*P* = 0.09, Figure 3). Liver mRNA expression of pyruvate dehydrogenase kinase, isozyme 4 (PDK4) was increased in animals consuming both the L-TRP and 5-HTP diets, but more significantly in 5-HTP fed dams (*P*<0.041, Figure 3). mRNA expression of PFK1 was only increased in the L-TRP group compared to CON (*P* = 0.001, Figure 3).

### Expression of Maternal Mammary Gland Glucose Transporters

**Figure 4:**
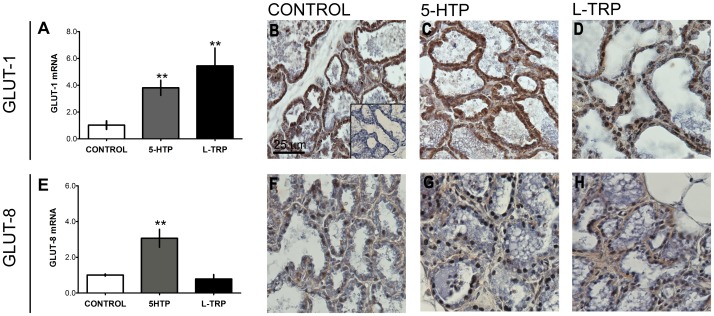
Effects of 5-HTP or L-TRP feeding on GLUT-1 and -8 expression in mammary gland of rats on day 9 of lactation. (A) GLUT-1 mRNA expression in the mammary gland of dams fed CON, 5-HTP, and L-TRP diets. GLUT-1 immunostaining in mammary glands collected on d9 of lactation from rats fed CON (B), 5-HTP (C), and L-TRP (D) diets. (E) GLUT-8 mRNA expressions in the mammary gland of dams fed CON, 5-HTP, and L-TRP diets. GLUT-8 immunostaining in mammary glands collected on d9 of lactation from dams fed CON (F), 5-HTP (G), and L-TRP (H) diets. Two asterisks (**) indicates statistical significance at *P*<0.01.

Entry of glucose into the mammary gland is an initial crucial step for the synthesis of milk lactose [28]. To date, GLUT-1 is thought to be the primary glucose transporter responsible for glucose uptake in the mammary gland, however several others have been detected in the mammary gland and little is known about their functions [28]. Mammary gland GLUT-1 mRNA expression was increased in dams consuming the L-TRP and 5-HTP diets (*P*<0.0082, Figure 4a). mRNA expression of GLUT-8 was increased in the mammary gland of dams consuming the 5-HTP exclusively (*P* = 0.023, Figure 4b). Antibody stains for GLUT-1 and -8 were positive in mammary tissue in animals consuming all three diets. While GLUT-1 was clearly located in epithelial cells (Figure 4b–4d), GLUT-8 appeared to be located in epithelial and stromal cells (Figures 4f–4h).

**Figure 5:**
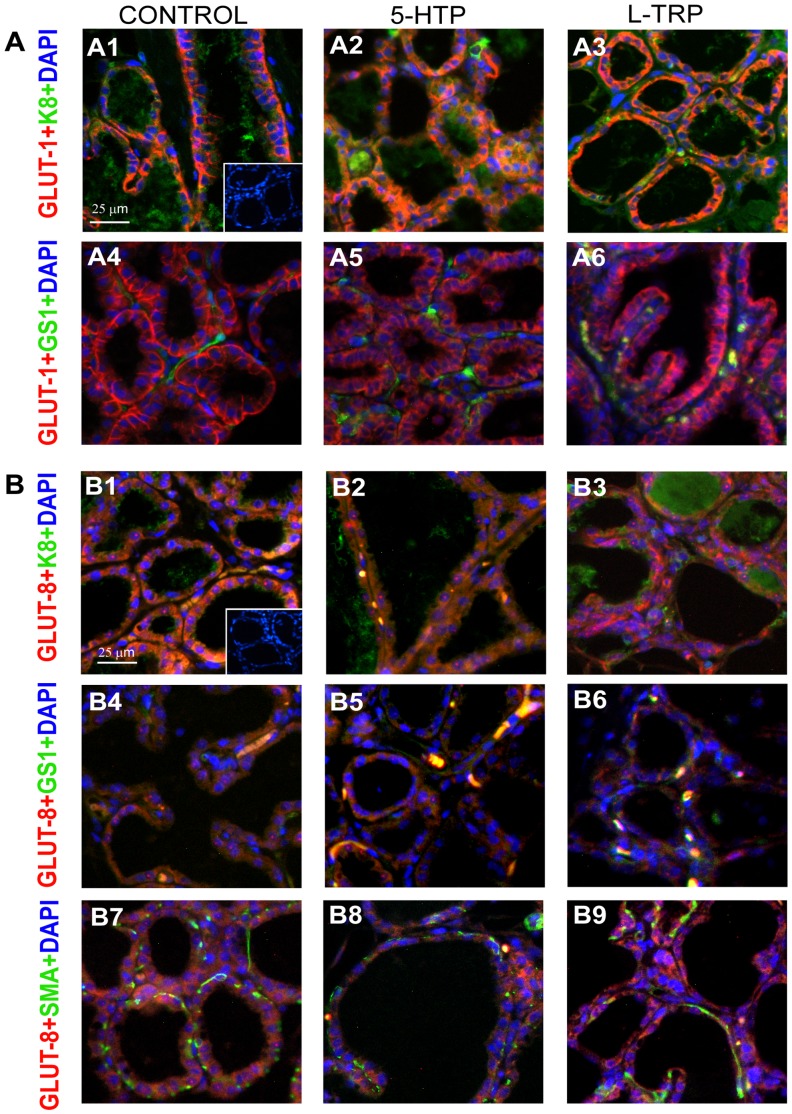
Fluorescent immunostaining of GLUT-1 and GLUT-8 in the mammary gland of dams fed CON, 5-HTP or L-TRP diets on day 9 of lactation. (A) GLUT-1 (red) and the luminal epithelial cell marker, keratin 8 (K8) (green, A1–A3), and the vascular endothelial cell marker, griffonia simplicifolia lectin 1 (GS1; green, A4–A6). (B) GLUT-8 (red) and K8 (green, B1–B3), GS1 (green, B4–B6), and the myoepithelial cell marker, smooth muscle actin (SMA, green, B7–B9). Cell nuclei for all sections were visualized with DAPI (blue). Inset contains a negative control (no primary antibodies).

Using immunofluorescence we demonstrated that GLUT-1 is present in K8+ mammary luminal epithelium (Figure 5 a1–a3) but not in GS1+ endothelium (Figure 5 a4–a6). Similarly, GLUT-8 was detected in K8+ mammary luminal epithelium (Figure 5 b1–b3) but was also detected in GS1+ endothelium (Figure 5 b4–b6) but not in SMA+ myoepithelium (Figure 5 b7–b9). Additionally, it appears that GLUT-1 and GLUT-8 in the 5-HTP group are not only present on the basolateral portion of the mammary epithelium, but also appear to be dispersed within the epithelia, possibly in specific organelles within the epithelial cells.

### pAMPK Activation in the Mammary Gland of Dams

**Figure 6:**
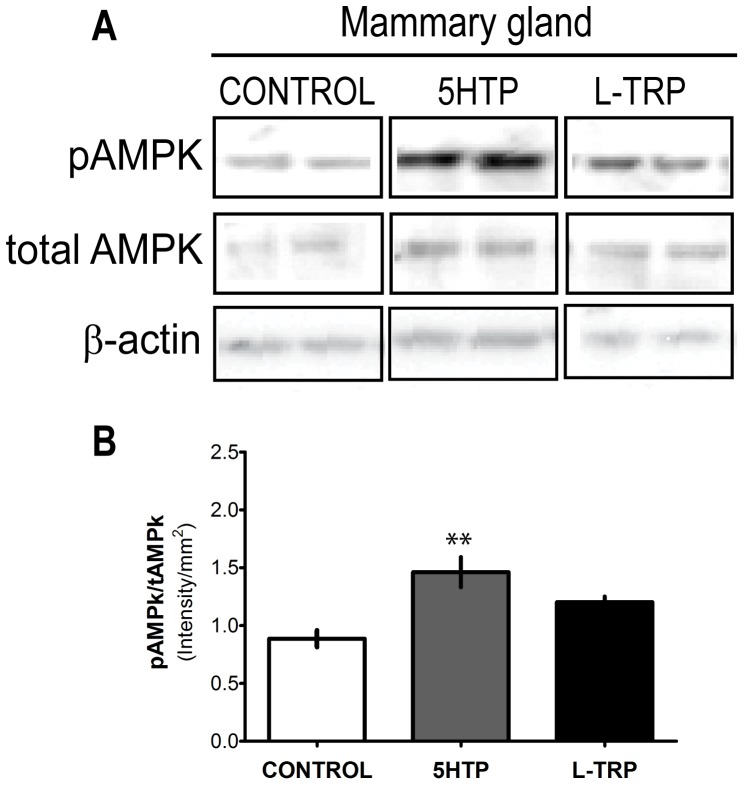
AMPK activation is increased in mammary glands of dams. Western blot analysis of mammary glands collected on d9 of lactation from dams fed CON, 5-HTP, and L-TRP diets. (A) Representative western blots for pAMPK, total AMPK and β-actin (n = 8 per group) (B). Two asterisks (**) indicates statistical significance at *P*<0.01.

AMPK is a known regulator of intracellular energy status, as well as uptake and metabolism of glucose in the mammary gland [29]–[30]. Therefore, we evaluated AMPK activation in the mammary gland in response to feeding dams supplemental 5-HTP and L-TRP. We assessed pAMPK activation by probing the (pAMPK/β-actin)/(tAMPK/β-actin) ratio using western blot analysis. 5-HTP animals had significantly increased pAMPK compared to CON and L-TRP animals (*P* = 0.0017, Figure 6 a, b). Expression of t-AMPK and β-actin remained constant between the 3 groups (Figure 6a).

## Discussion

Here, we report that feeding supplemental 5-HTP and L-TRP to dams during the transition from pregnancy to lactation increases circulating 5-HT. Dietary 5-HTP caused the most pronounced, immediate and sustained increase in 5-HT, in contrast to dietary L-TRP, which did not result in increased 5-HT until the final day of the experiment. It is likely that the L-TRP group took more time to increase circulating 5-HT, in contrast to 5-HTP, which is exclusively converted to 5-HT, L-TRP is utilized for synthesis of 5-HT as well as numerous other compounds [7], [31]. 5-HT levels were increased in the mammary gland and liver of both treatment groups on d9 of lactation, which is in accordance with serum 5-HT levels at this time. Additionally, we saw increased TPH1 mRNA expression in mammary gland and liver of the 5-HTP cohorts, suggesting positive feedback stimulation of 5-HT. The rapidly upregulated and sustained 5-HT synthesis in the 5-HTP group compared to the L-TRP group suggests that there are other possible mechanisms by which dietary L-TRP was regulating maternal energy metabolism, independent of 5-HT and this could be responsible for the differences in the effects between feeding the two diets.

Hepatic glucose synthesis during late pregnancy and early lactation must increase to accommodate uterine and mammary demands, respectively [32]. In mice, it has been reported that 5-HT is important in the regulation of metabolic homeostasis and there are at least two different effects it has on glucose metabolism [3], [17], [33]. 5-HT has been determined to induce hypoglycemia and hyperinsulinemia, as well as hyperglycemia due to interaction with different 5-HT receptor subtypes that are present within the liver [3], [17], [33]. In our study, we saw no differences in serum insulin concentrations in dams fed supplemental 5-HTP or L-TRP, also animals had ad libitum access to feed so any possible meal effects on insulin concentration were not accounted for. Interestingly, serum and milk glucose concentrations were lower in the L-TRP group compared to the 5-HTP and CON groups at all times evaluated. It is plausible that 5-HT is not ultimately responsible for decreased glucose concentrations in the circulation and milk, as L-TRP did not increase maternal circulating 5-HT until d9 of lactation. One possible explanation could be that L-TRP is involved in numerous other metabolic synthesis pathways [31]. Although serum and milk glucose concentrations were decreased in the L-TRP group, there was not a decrease in milk lactose concentrations, suggesting that there was sufficient glucose for milk lactose synthesis. In fact, only 10–20% of glucose supplied to the lactating rat mammary gland is used to produce lactose [34]. An additional possibility could be that there is an effect on milk fat synthesis as glucose is a critical contributor to milk fat in non-ruminant animals [1], [2]. However, we did not measure milk fat percent within our milk samples. Our results support the notion that feeding 5-HTP to dams during the transition from pregnancy to lactation increases 5-HT levels in the circulation, liver and mammary gland and allows dams to maintain serum and milk glucose and lactose independently of insulin.

Multiple glucose transporters are present in mammary gland tissue, including GLUT-1, -8, -12 (energy independent glucose transporters, solute carriers, SLC2) and SGLT1 (sodium-dependent transport, mediated by the sodium/glucose co-transporters, SLC5), and the concentration of these transporters increases at the onset of lactation [35]–[36]. During pregnancy, rat GLUT-1 abundance increases progressively, peaks during mid-lactation, and then tapers within 24 h of weaning [37]. Rat GLUT-1 was previously localized to mammary epithelial cells [38] and we confirmed this observation in dams, regardless of dietary supplement. The GLUT-1 transporter localizes to the basolateral surface of mammary gland epithelial cells at peak of lactation [37]–[39] and is present in the golgi membrane during late pregnancy and lactation [40]. In dams receiving the supplemental 5-HTP, GLUT-1 was primarily located in the basolateral membrane but was also distributed in intracellular vesicles. We speculate that intracellular GLUT-1 in the 5-HTP cohort may indicate GLUT-1 targeting to the golgi apparatus, which is critical to lactose synthesis [40].

GLUT-8 (25% sequence identity to GLUT-1) [41] is highly expressed in testis, and is implicated in fertility and energy metabolism of sperm cells [42]. GLUT-8 also mediates glucose uptake in Xenopus oocytes [43] and is present in kidneys, liver and the mammary gland [35]. To date, little is known about the role of GLUT-8 in the mammary gland during lactation. GLUT-8 mRNA expression was increased in the mammary gland of the dams receiving supplemental 5-HTP. It is possible that GLUT-8 is important for the uptake of glucose in the mammary gland and potentially for the synthesis of lactose. Additionally, we identified that GLUT-8 was present in mammary epithelial cells as well as stromal cells, specifically in the vascular endothelium between alveoli. GLUT-8 expression has been reported in brain endothelial cells under conditions of metabolic stress [44]. A possible explanation for the role of GLUT-8 in the mammary gland could be to regulate glucose transport from vasculature to the mammary gland, in addition to regulation within the mammary gland itself. This is supported by the fact that we observed GLUT-8 to localize not only on the basolateral surface of the epithelium, but also intracellularly. Most GLUT-8 is not present at the cell surface, and co-localization with both the endoplasmic reticulum and late endosomes/lysosomes has been previously reported [41]. To our knowledge, this is the first study to demonstrate the localization of GLUT-8 within the mammary gland and its potential to distribute to different locations within the mammary epithelium.

In our study, supplemental 5-HTP and L-TRP increased hepatic mRNA expression of PDK4 on d9 of lactation. PDK4 inhibits the pyruvate dehydrogenase complex reducing the conversion of pyruvate to acetyl-CoA, thereby conserving glucose [45]. It has been shown that mice lacking PDK4 have decreased blood glucose levels compared to wild type mice [45]. 5-HTP also induced expression of PC, which catalyzes pyruvate carboxylation into oxaloacetate in the gluconeogenic pathway, and PCK1, which converts oxaloacetate to phosphoenolpyruvate [45]–[46]. Increased mRNA expression of PC, together with PCK1 in the liver of 5-HTP dams, suggests the possibility that 5-HT may be important for regulating gluconeogenesis through the regulation of these enzymes [46]. Interestingly, mRNA expression of hepatic PFK1, the most important and tightly regulated enzyme of in glycolysis [47], was increased in only the L-TRP fed dams. Based on the fact that dams fed L-TRP did not have elevated circulating 5-HT until d9 of lactation, we cannot definitively attribute this result to increased 5-HT concentrations. It has been previously reported that 5-HT increases PFK in the liver of mice, however we saw no increases in the mRNA expression of 5-HTP fed dams [21]. Our results indicate that supplemental L-TRP was potentially not only increasing gluconeogenic enzymes, but also glycolytic enzymes. These findings might explain the differences in serum glucose observed between the 5-HT and L-TRP groups, as serum glucose was significantly reduced in the L-TRP fed dams. Overall, our results demonstrate an increase in hepatic gluconeogenic enzymes in dams fed supplemental 5-HTP, and increases in both gluconeogenic and glycolytic enzymes in dams fed supplemental L-TRP. Our results suggest the possibility that 5-HT may be involved in regulating hepatic energy metabolism during the transition from pregnancy to lactation, however more extensive studies need to be performed.

5-HT is involved in regulation of metabolic homeostasis [3]. Due to the effects of increasing 5-HT during the transition from pregnancy to lactation on markers of energy metabolism in the liver and glucose transporters in the mammary gland, we thought it pertinent to investigate the effects of 5-HT on AMPK in the mammary gland. We showed that phosphorylation of AMPK, a crucial cellular energy sensor, is increased in mammary glands of dams fed supplemental 5-HTP. Upon activation, AMPK is responsible for decreasing activity or expression of catalytic proteins, which conserves ATP by switching off biosynthetic pathways [29]–[30]. Atypical antipsychotics have been demonstrated to increase pAMPK, and the mechanisms governing this response are partially mediated by 5-HT [48]. AMPK also regulates metabolic energy balance at the whole body level and regulates uptake and metabolism of glucose and fatty acids [30]. Interestingly, AMPK promotes glucose uptake into cells that express GLUT-1 [29]. It is possible that AMPK could be responsible for the increase of GLUT-1, and potentially GLUT-8, in the mammary epithelium of dams fed supplemental 5-HTP. Furthermore, 5-HTP up-regulation of AMPK could be important for milk fat synthesis as well, however we did not measure milk fat in this study.

### Conclusion

Energy homeostasis is critical during the transition from pregnancy to lactation in mammals. Herein, we demonstrate that dietary administration of 5-HTP to rat dams transitioning from pregnancy to lactation enhances 5-HT production, affects hepatic energy metabolism and significantly impacts mammary gland glucose transporters. Dietary L-TRP also enhances peripheral 5-HT production, though this response is delayed compared to dietary 5-HTP, and it is likely that the effects seen in the L-TRP group were due to an alternative mechanism. We demonstrate for the first time the presence of GLUT-8 in rat mammary epithelial and endothelial cells, which supports the potential for this transporter to be involved in regulating glucose transport from the vasculature to the mammary epithelium during lactation. Finally, we showed that dietary administration of 5-HTP is capable of impacting hepatic energy metabolism. Further research is necessary to describe the role of 5-HT in regulating energy metabolism within the liver and glucose transport within the mammary gland during the transition from pregnancy to lactation as well as the potential effect on milk fat synthesis.
